# Comparison of Physical Therapy with Energy Healing for Improving Range of Motion in Subjects with Restricted Shoulder Mobility

**DOI:** 10.1155/2013/329731

**Published:** 2013-11-14

**Authors:** Ann Linda Baldwin, Kirstin Fullmer, Gary E. Schwartz

**Affiliations:** ^1^Laboratory for the Advances in Consciousness and Health, Department of Psychology, University of Arizona, Tucson, AZ 85721-0068, USA; ^2^Department of Physiology, College of Medicine, University of Arizona, Tucson, AZ 85724-5051, USA

## Abstract

Two forms of energy healing, Reconnective Healing (RH) and Reiki, which involve light or no touch, were tested for efficacy against physical therapy (PT) for increasing limited range of motion (ROM) of arm elevation in the scapular plane. Participants were assigned to one of 5 groups: PT, Reiki, RH, Sham Healing, or no treatment. Except for no treatment, participants were blinded as to grouping. Range of Motion, self-reported pain, and heart rate variability (HRV) were assessed before and after a 10-minute session. On average, for PT, Reiki, RH, Sham Healing, and no treatment, respectively, ROM increased by 12°, 20°, 26°, 0.6°, and 3° and pain score decreased by 11.5%, 10.1%, 23.9%, 15.4%, and 0%. Physical therapy, Reiki, and RH were more effective than Sham Healing for increasing ROM (PT: *F* = 8.05, *P* = 0.008; Reiki: *F* = 10.48, *P* = 0.003; RH: *F* = 30.19, *P* < 0.001). It is possible that this improvement was not mediated by myofascial release because the subjects' HRV did not change, suggesting no significant increase in vagal activity. Sham treatment significantly reduced pain compared to no treatment (*F* = 8.4, *P* = 0.007) and was just as effective as PT, Reiki, and RH. It is the authors' opinion that the accompanying pain relief is a placebo effect.

## 1. Introduction

Shoulder pain is a common musculoskeletal symptom, accounting for 16% of all musculoskeletal complaints [1]. Lifetime prevalence of shoulder pain has been reported to range from 7% to 36% of the population [2]. The precise causes of shoulder pain within the joint are unknown, but the nongenetic variants are thought to result from joint intra-articular degeneration (osteoarthritis), structural damage (torn rotator cuff and/or ligaments), infection or inflammation (bursitis and tendinitis), or arthritis [3–6]. Rotator cuff pathology and subacromial impingement are among the most common diagnoses pertaining to shoulders [2, 7].

Apart from pain, shoulder damage or degeneration often leads to limited ROM of the shoulder. Chronic soft tissue disorders, such as tendinitis, bursitis, rotator cuff tears, and impingement syndrome, may result in secondary adhesive capsulitis. It is the adhesions that limit ROM [8]. Physical therapy is usually the first choice of treatment for these problems [9]. Physical therapy encompasses many types of interventions including manual manipulation, therapeutic exercise, functional training, and electrotherapeutic modalities. However, it is the manual manipulation that requires the most sustained physical effort by the therapist. In a study addressing job strain in physical therapists [10], 58% of the 882 physical therapists interviewed experienced a work-related ache or pain during the year prior to the follow-up survey. The most common region was the low back, followed by the wrist and hand. In another investigation [11], 83% of 344 physical therapists who returned completed questionnaires indicated that they were treating or handling a patient when they first experienced lower back pain on the job. When asked to select the mechanism of injury, 24% of the respondents selected “lifting with sudden maximal effort” and 24% chose “bending and twisting.” Over half of these therapists reported recurrent episodes. Since manual manipulation may cause recurrent pain for the therapists it would be advantageous to limit its use to cases in which it is strictly necessary in order to promote a patient's recovery.

The purpose of this research was to determine whether manual manipulation is necessary for success in short-term treatment of limited ROM of arm elevation in the scapular plane. Other therapies, such as Reiki and Reconnective Healing, that do not involve physical effort were compared with PT for effectiveness in improving ROM. The 2007 National Health Interview Survey, compiled by Barnes et al., [12] indicated that 1.2 million adults and 161,000 children in the United States received one or more sessions of energy healing, such as Reiki, during the previous year. According to the American Hospital Association, [13] 15% of American hospitals (more than 800 facilities) offered Reiki as a hospital service in 2007. A joint publication by the American Holistic Nurses Association and American Nurses Association [14] lists Reiki as an accepted form of treatment.

Reiki is administered by the hands, placed lightly on or near the body of the recipient. The Reiki practitioner focuses his attention on the recipient and then allows the energy to flow passively through their body and hands where it is passed to the recipient. These procedures are described by various Usui Reiki training manuals and Reiki websites, such as http://www.reiki.org/FAQ/Questions&Answers.html; http://www.reiki.org/reikipractice/practicehomepage.html#Intention.

Practitioners who learn Usui Reiki receive similar training and specific objectives are laid out for each one of the three levels (e.g., see http://www.reiki.org/Download/FreeDownloads.html).

Peer-reviewed research demonstrates that recipients of Reiki experience feelings of relaxation, mental clarity, pain relief, decreased anxiety, and a sense of wellbeing. These studies are described in several reviews [15–17]. Physiological signs of relaxation in recipients include increases in their parasympathetic autonomic nervous system activity [15, 18–20]. There are currently no studies published in peer-reviewed journals addressing the effects of Reiki on ROM.

Reconnective healers work with their hands to sense and manipulate what they term as biofields, which are energy fields that surround living beings [21]. Reconnective Healing is said to involve tuning into the healing energy frequencies needed by each recipient and receiving and sensing the energy. Unlike Reiki, there is no specific “centering” or “grounding” process involved in which practitioners focus on the present moment through concentrating on their breathing. All Reconnective healers receive training from instructors who have followed a prescribed syllabus which is the same worldwide, and so the procedure is reproducible among healers (see http://www.thereconnection.com/programs/reconnective-healing-level-i-ii/).

People receiving Reconnective Healing anecdotally report a range of sensations including warmth, tingling, cold, and throbbing and physiological responses such as rapid eye movements, deepening breath, stomach gurgling, and muscle twitching. However, there are no published studies addressing the effectiveness of RH in reducing pain. Overall, none of the energy healing modalities have been tested for efficacy on people with limited ROM.

In this study, manual manipulation (joint mobilization, long axis distraction, and gentle rebounding) was tested against RH and Reiki in patients with limited ROM. These energy healing therapies only involve light touch or no touch and so if they are as effective as manual manipulation in improving ROM, this would imply that manual manipulation, per se, is not necessary for alleviating this particular impairment.

Another objective was to assess self-reported pain and heart rate variability (HRV) as secondary outcome variables, before and after each type of treatment; HRV is a measure of sympathovagal balance which may provide some insight into possible mechanisms of pain relief because it is known that stimulation of the vagus nerve can reduce pain [22]. Such stimulation can occur directly through myofascial release [23] and there is some evidence that it can be mediated indirectly by application of Reiki [20, 24].

## 2. Methods

### 2.1. Recruitment and Consenting of Participants

This investigation was approved by the University Institutional Human Subjects Protection Committee. People with limited range of motion of one or both shoulders were recruited for the study by providing fliers to local chiropractors, physical therapists, masseurs, and fitness coaches, by posting the flier at various locations of the university campus such as Campus Health Service, Family and Community Medicine, Student Recreation Center, Libraries, Arthritis Center, Athletics Departments, and Center for Integrative Medicine and by running a radio advertisement on National Public Radio. The fliers were deliberately posted in a wide variety of locations in order to attract potential participants who were representative of the population at large rather than just individuals looking for nontraditional therapies. Investigators' conversations with the potential participants during the enrollment process confirmed that most potential participants were fairly traditional in their medical choices.

Potential participants were first screened by telephone to determine whether they met the following inclusion and exclusion criteria:

1. at least 18 years of age;
2. self-ambulatory (no assistive devices);
3. having had a nongenetic ROM limitation for at least one year and having some form of medical documentation of the problem;
4. ROM limitation being the result of injury (sports related or otherwise), surgery, arthritis, or adhesive capsulitis;
5. having had no experience of energy healing (Reiki or Reconnective Healing), including sessions, seminars, or reading “The Reconnection” by Dr. Pearl [25];
6. if female, must not be pregnant.

Those who met all the criteria were interviewed at the university and tested to determine whether the ROM of at least one of their arms was limited to somewhere between 30° below the horizontal plane and 60° above the horizontal plane. People who were qualified were consented for the study after the experimental protocols; risks and benefits had been explained. During the consenting process, participants filled out a demographics questionnaire, consisting of birthplace, ethnicity, age, height, and weight. They also provided the following information regarding their specific shoulder problem:

1. reason for ROM limitation and whether diagnosed by a physician;
2. length of time they have had limited ROM;
3. whether left, right, or both arms are affected;
4. types of health-based practitioners the person had previously visited for this * *problem, such as medical doctor (MD), chiropractor (DC), osteopath * *(DO), physical therapist (PT), naturopath (ND), acupuncturist (LAc), and massage therapist;
5. whether or not the problem was diagnosed as a result of MRI scanning or radiographs;
6. whether the subject had surgery to attempt to alleviate the problem, and if so, the type of surgery. Was internal fixation inserted? Did surgery make the problem better/worse/or cause no change?
7. Whether a prior surgery caused the problem.

### 2.2. Group Assignment

A power analysis at 80% power was performed to find the number of subjects necessary to detect a significant difference between groups of 20° for arm elevation in the scapular plane, based on the previously observed variance. From this test, a group size of 15 was chosen for the study. The value of 20° was chosen because the focus of this study was to determine whether energy healing produced large improvements in ROM. The study was conducted in the following way: six experimental sessions, involving 12–17 participants, were held at a local hotel, easily accessible by participants and therapists. The six sessions were carried out in the following order: (i) Reconnective Healing, (ii) Reiki, (iii) Sham Healing, (iv) physical therapy, (v) control, and (vi) RH plus PT. Due to logistics, recruitment was performed in two phases. In the first phase subjects were recruited for the RH, Reiki, and Sham Healing groups, and in the second phase they were recruited for the RH, PT, and control groups. In both cases participants were assigned to one of three groups on a rotating basis according to order of recruitment.

### 2.3. Demographics of Experimental Participants

**Figure 1:**
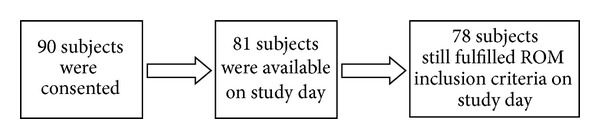
Flow chart of subject participation.

A flow chart of the retention of 90 recruited subjects is shown in Figure 1.

Of the 78 participating subjects, 37 were males and 41 were females. All subjects were Caucasian, except for seven Hispanic, two Asian, and one “other.” The participants' ages ranged from 20 to 89 and the mean age for each group was 61.3 ± 53.2 (SD) (control), 58.5 ± 59.4 (Sham), 64.4 ± 56.6 (PT), 66.4 ± 58.3 (Reiki), and 59.8 ± 52.2 (RH). The difference in mean age between groups was not statistically significant (ANOVA analysis of variance). The corresponding gender ratios for each group (male to female) were 1.14, 0.50, 0.60, 1.50, and 1.12.

### 2.4. Medical History and Diagnosis of Experimental Participants

The diagnoses of the participants relevant to restricted shoulder mobility are shown in Table 1. In most cases (55/78) the diagnoses were made using radiographs and/or magnetic resonance imaging. The frequency of the different diagnoses was fairly uniform between groups (see Table 2). Nineteen of the participants had experienced their condition for less than two years, 30 for between two and five years, and 29 for more than five years. Sixteen of the participants had experienced shoulder surgery, between one and 20 years previously, in an attempt to improve their range of motion and/or reduce pain. The surgery had been partially successful for a short time in five cases and unsuccessful in the others. Each experimental group included some participants who had had surgery (Table 2).

### 2.5. Selection of Therapists

Three therapists were selected for each arm of the study, except for the no-treatment control group. Each therapist would work on one-third of the participants in their group. The Reconnective healers (2 males, 1 female) were experienced instructors from The Reconnection LLC Teaching Team, who train students worldwide. The Reiki practitioners (1 male, 2 females) were local, had been practicing Usui Reiki professionally for a minimum of 4 years, were 6 generations removed from the founder, Mikao Usui, and had received the highest level of Reiki training. The licensed physical therapists (3 females) were local and did not include energy work in their repertoire. All had practiced PT for over 10 years, had their own practices, and were experienced in treating complex medical and physical conditions in a range of traditional PT settings. Three people (1 male, 2 females) who had absolutely no experience with any form of energy healing were chosen to be sham healers.

### 2.6. Instructions to Therapists

All practitioners were instructed not to disclose what type of therapy they would provide because the participants did not know what kind of treatment they were getting.

Reconnective healers used mainly hands-off treatment. Reiki practitioners focused their healing intention on increasing the participant's ROM and used mainly hands-on treatment for each participant. Physical therapists were asked to give their normal basic manipulation of the shoulder joints and surrounding deep tissue. The physical therapists chose to provide gentle passive ROM and simple glenohumeral joint mobilizations (inferior glides) in attempt to increase shoulder abduction and flexion. Long axis distraction was applied to the glenohumeral joint, as was gentle rebounding. Sham healers were asked to wave their hands slowly over the participant's shoulder area and upper body, 6–12 inches away from their body, and to occasionally draw their hands back away from them, similar to the actions of Reconnective Healers.

All therapists were told that the participants had ROM limitations resulting from shoulder injuries or arthritis but were not told any details about the specific problems of individual participants or which shoulder was most affected. Each practitioner worked with 2 participants, one after the other, and then had a 20-minute rest before working with the next 2 participants. During the practitioner rest breaks, the participants went to another room for pre-or post measurement of their ROM and HRV.

### 2.7. Experimental Procedure

On arrival, participants were informed by a student that they would be receiving a treatment, which may be energy healing, Sham Healing, or PT, or no treatment at all, to assess how this affects their range of motion. The student was blinded as to the type of therapy each participant would receive except for those in the no-treatment control group. The participants were similarly blinded. Each participant filled out a visual analog scale (VAS) expectancy survey, asking whether they expected the treatment to work. They were then shown a video explaining how their ROM would be measured before and after treatment. Briefly, they would be asked to stand close in front of a wall, without touching it, with their arms at their sides. They would then be video-recorded as they moved their arms out to the sides and then up towards their head, in a scapular plane (i.e., not bringing their arms forward) as far as they could go, while keeping their arms straight, so as not to involve the elbow joint in the exercise, once with palms facing up and once with palms facing down. One reason ROM was measured by video analysis rather than using a goniometer is that it is noninvasive. A goniometer is positioned on the subjects, scapular spine as they hold their full ROM, which can be painful. In addition, since we were looking for large improvements in ROM, there was no need for the 0.1° accuracy of the goniometer.

After watching the video, each participant performed the exercise and then filled out a VAS pain assessment reflecting the maximum pain they felt when moving their arms. Next, each participant was seated for measurement of HRV. It is generally recognized that respiration has an important effect on HRV and so respiration was also measured in this study. A strap was snugly placed around their chest to measure respiration rate, and a pulse sensor was connected to the middle finger of their left hand to measure pulse rate (interbeat interval) for calculation of HRV. The strap and sensor were connected to a computer via a BioGraph Infiniti ProComp module (Thought Technology Ltd., Montreal, Canada) to enable data recording. Each participant was asked to relax, keep still, and not speak for five minutes while data were recorded.

Next, each participant was taken to the treatment room to meet the therapist or to lie supine on a massage table for 10 minutes if they were in the no-treatment control group. In this case, since no therapist was present, a student sat quietly in the same room as the participant and then told them when the 10 minutes was up and directed them back to the measurement room. If a therapist was present, he asked the participants to show how high they could raise their arms in a scapular plane out to the sides and towards their head, keeping their arms straight and palms down, and took a photo of them showing their maximal ROM with a camera that was provided in the treatment room. Then he asked the subjects to lie supine on the massage table for the treatment. After the therapist had completed the treatment he asked the participants to stand and demonstrate their ROM, palms down as before, and took another photo. These photos were later compared with the videos taken in the measurement room to check for reproducibility of pre-and postmeasures. The participant was then guided to the measurement room to reassess his ROM, pain evaluation, and HRV.

### 2.8. Outcome Measures

The primary outcome variable was pre-and postmeasurement of ROM of arm elevation in the scapular plane. The video measure of ROM was highly reproducible. Corresponding measures for a given person taken in the measurement and treatment rooms only differed by an average of 2°. Secondary outcome variables were (i) expectancy, pretreatment, that the treatment would work, (ii) self-reported pain level during elevation, and (iii) HRV. Pain and HRV were evaluated pre- and posttreatment or no treatment. All data were coded to conceal the identity of each subject and their experimental group from the data analyzer, thus minimizing, and hopefully preventing, effects of possible researcher bias.

#### 2.8.1. Range of Motion

**Figure 2:**
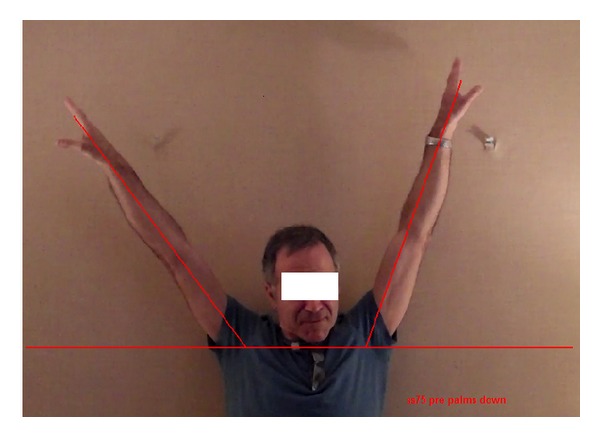
Depiction of humeral angle as a measure of range of motion. Angles above the horizontal are positive from 0° to 90°, and those below are negative.

The video recordings were used to obtain an image of each participant's maximal ROM pre- and posttreatment. From each image the angle of elevation of each arm (as depicted by the straight line connecting the wrist to the mid-point of attachment of the shoulder to the trunk) could be measured above or below the horizontal (humeral angle). A depiction of this measurement is shown in Figure 2. Angles above the horizontal were positive from 0° to 90°, and those below the horizontal were negative. Four angles were obtained for each pre- and postmeasure: left arm palms up, left arm palms down, right arm palms up, and right arm palms down.

#### 2.8.2. Secondary Outcomes

- Expectancy that the treatment would work was assessed with a 100 mm VAS before the subject entered the treatment room. Each subject was asked to mark a vertical line on the VAS to indicate expectancy. No expectancy at all was represented by 0 mm and definite expectancy by 100 mm.
- Pain severity was assessed with a 100 mm VAS. Each subject was asked to mark a vertical line on the VAS to indicate perceived level of pain. No pain was represented by 0 mm and extreme pain by 100 mm. A previous study [26] performed on patients treated for rotator cuff disease indicated that the minimal clinically important difference (MCID) for VAS measuring pain is 14 mm.
- Interbeat interval (heart rate) data, measured over a period of 5 minutes pre- and posttreatment, were exported as a text file from the BioGraph Infiniti Physiology Suite software into a freeware HRV program, http://kubios.uef.fi/. This program analyzes the data to quantify the variability in heart rate that exists in a given recording in terms of established measures. Time domain parameters include the standard deviation of the interbeat interval (IBI), SDRR, which provides a gross measure of HRV, and the root mean square of successive differences in IBI (RMSSD), which reflects the parasympathetic activity of the autonomic nervous system.

### 2.9. Statistics

Four-way Repeated Measures Analysis of Variance (ANOVA) was run for ROM to test for significant differences among the 5 groups for the treatment effect, pre versus post, palms down or up and left arm or right arm. If the difference was significant, ANOVA was then repeated pairwise. Similar tests were run for pain scores, HR, and HRV. STATISTICA for Windows software was used for the analysis.

## 3. Results

### 3.1. Expectation

There was no significant difference between the average self-reported expectation levels of the 5 groups (*F* = 0.25, *P* = 0.9). The mean expectation values were as follows: control: 54.5; Sham: 64.1; PT: 56.5; Reiki: 57.8; and RH: 55.1.

### 3.2. Range of Motion

**Figure 3:**
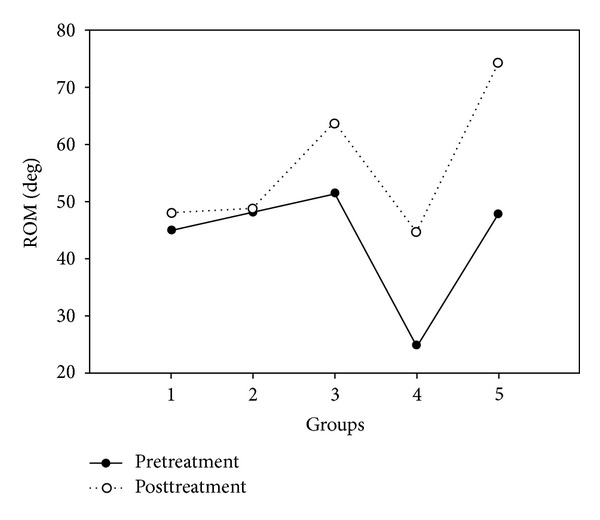
Average range of motion, in degrees above the horizontal, for all 5 treatment groups.

Most of the patients who received PT, Reiki, or Reconnective Healing showed improved ROM. Relative numbers of subjects in each group showing improvement were as follows: PT: 15/16; Reiki: 12/15; and RH: 16/17. Although it appears that the Reiki group started the study with a lower range of motion than the other groups, this difference was not statistically significant. There was no significant difference between the pretreatment ROM measures of the 5 groups (ANOVA analysis of variance, *F* = 1.4,   *P* = 0.24). The average pretreatment ROM values for all 5 groups were positive, although some individuals showed negative values. Comparing postmeasures to premeasures there was a highly significant difference between the 5 groups (averaged over palms up and down and left and right arms, *F* = 10.3,   *P* < 0.001). These results are shown in Figure 3 and Table 3.

On average ROM increased by 3°, 0.6°, 12°, 20°, and 26° for control, sham, PT, Reiki, and RH groups, respectively. Pairwise analysis showed that Sham treatment was no better than the no-treatment control and that PT, Reiki, and RH were all significantly more effective than Sham (PT: *F* = 8.05,   *P* = 0.008; Reiki: *F* = 10.48,   *P* = 0.003; RH: *F* = 30.19,   *P* < 0.001). Reconnective Healing was significantly more effective than PT (*F* = 9.61,   *P* = 0.004), but there was no significant difference between Reiki and PT (*F* = 1.73,   *P* = 0.20).

### 3.3. Self-Reported Pain

**Figure 4:**
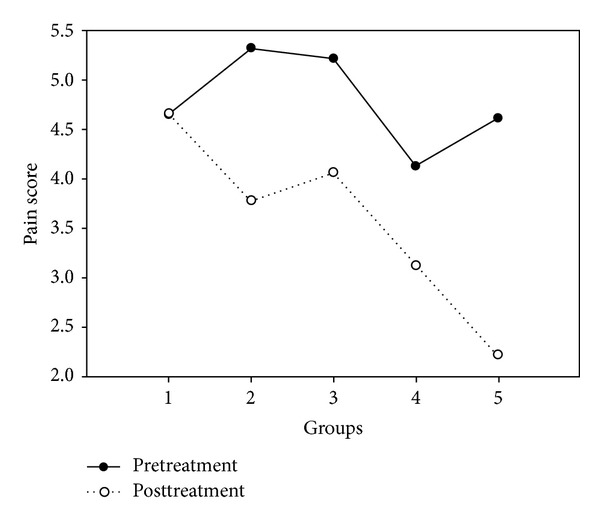
Average pain score (VAS) for all 5 treatment groups.

There was no significant difference between the pretreatment pain scores of the 5 groups (ANOVA analysis of variance, *F* = 0.73, *P* = 0.57). Comparing postmeasures to premeasures there was a highly significant difference between the 5 groups, (*F* = 4.75,   *P* < 0.002). These results are shown in Figure 4 and Table 4.

On average the pain score decreased by 0%, 15.4%, 11.5%, 10.1%, and 23.9% for control, sham, PT, Reiki, and RH groups, respectively. However, the average pain reduction in the PT and Reiki groups did not reach the MCID. Pairwise analysis showed that unlike the ROM results, the sham treatment was significantly more effective in reducing pain than the no-treatment control (*F* = 8.4,   *P* = 0.007); in fact, none of the other treatments were anymore effective than the sham treatment (PT: *F* = 0.42,   *P* = 0.52; Reiki: *F* = 0.57, *P* = 0.46; RH: *F* = 1.9,   *P* = 0.18). Although RH was no more effective than the sham treatment in reducing pain, pairwise comparisons indicated that RH was more effective than Reiki (*F* = 4.77,   *P* = 0.037) or PT (*F* = 5.48,   *P* = 0.026).

### 3.4. Heart Rate

**Figure 5:**
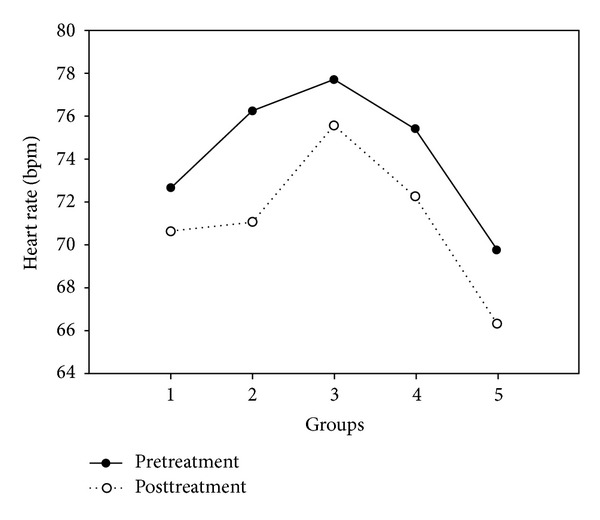
Average heart rate (beats/minute) for all 5 treatment groups.

Although heart rate significantly decreased posttreatment when all 5 groups were considered (*F* = 6.55,   *P* = 0.01), there was no difference in this reduction in HR between the groups, including the no-treatment control group. The results are shown in Figure 5.

### 3.5. Heart Rate Variability

The mean respiration rate of participants did not vary between groups. Therefore, the HRV results of the study were not influenced by alterations in respiration. Neither SDRR nor RMSSD significantly changed posttreatment compared to pretreatment when all 5 groups were considered (SDRR: *F* = 2.3,   *P* = 0.134; RMSSD: *F* = 1.46,   *P* = 0.23).

## 4. Discussion

This study showed that a 10-minute session of RH or Reiki was as effective as PT in improving ROM in people with restricted shoulder mobility; in fact RH, but not Reiki, was significantly more effective than PT when performed for this short time period. These results cannot be explained by a placebo effect because sham treatment did not significantly improve ROM. On the other hand, although PT, RH, and Reiki all significantly reduced the pain scores reported by participants compared to no treatment, the sham treatment was just as effective as the 3 healing modalities. The reduction in pain experienced by participants apart from those in the no-treatment group can be attributed to the placebo effect.

It is interesting that Sham Healing significantly reduced pain but did not improve ROM. These results suggest that the beneficial effects of Reiki and RH (but not Sham) on ROM may arise from alterations in local joint or muscle structures rather than the pain system. The success of the Sham Healing in reducing pain was probably triggered by the expectation of healing arising from the appearance and actions of the sham healer that may have then stimulated a release of endogenous opiates or activated the dopaminergic system [27].

Previous experiments evaluating the immediate effectiveness of PT (manual manipulation only) in improving ROM and reducing pain in subjects with shoulder problems report mixed results and small sample sizes. Surenkok et al. [28] showed that scapular mobilization of the affected shoulder of 13 people with painful shoulder restriction significantly improved ROM by an average of 4°. The mobilization included superior and inferior gliding, rotations, and distraction to the scapular. A control group was included. However, there was no significant reduction of pain as measured by a VAS when participants raised their arms before and after treatment. Teys et al. [29] tested a Mulligan's mobilization with movement technique, in which the physical therapist applies a sustained glide to the glenohumeral joint while the patient concurrently actively moves the joint, on 8 patients with painful shoulder constriction. Sets of 10 repetitions were applied with a 30 s rest interval between sets. A control group was included. This type of therapy had an immediate positive effect on both ROM and pressure pain threshold. Range of motion increased on average by 15.3°. Pressure pain threshold, or the degree of pressure sufficient to cause the patient pain when applied to the most sensitive point on the anterior aspect of the shoulder, was significantly decreased by 20%. However, the change in ROM was not related to the reduction in pain pressure threshold, consistent with our finding that Sham Healing significantly reduced pain but did not affect ROM.

Five other studies investigated the effects of PT manual manipulation on patients with painful shoulder restriction, but these experiments extended over weeks and no measurements were reported after the first session. Three of the 5 investigations showed improvement in average ROM after treatment [30–32]. Two studies did not show improvement [33, 34] but only mid-range rather than endrange manipulations were applied to the shoulders.

The mechanisms by which PT, Reiki, and RH improved ROM are not known. A theoretical basis for the action of manual manipulation PT and its effect in the body has been advanced based on autonomic activation causing concomitant vasodilatation, smooth muscle relaxation, and increased blood flow, resulting in improved ROM, decrease in pain perception, and/or change in tissue. In support of this theory it was shown that cervical myofascial release, such as that used by physical therapists, shifts sympathovagal balance from sympathetic to parasympathetic [23]. However, the improvement in ROM seen in the PT group in the current study may not have been mediated by this mechanism because no significant increase in HRV was observed after PT. For the same reason the beneficial effects of RH and Reiki in this case did not seem to operate through rebalancing of the autonomic nervous system.

One limitation of this study is that inferences drawn from the results should be confined to those seen in a single 10-minute treatment session with no follow-up. Another possible limitation is that the physical therapists chose to only include manual therapy performed at the glenohumeral joint rather than the entire shoulder complex and this may have limited their effectiveness. Further studies to evaluate such issues as the timecourse of the effect of PT, Reiki, and RH and the outcome on disability and function are warranted. There is a clear clinical need for nonsurgical treatments that are safe and effective for chronic, painful shoulder.

## 5. Conclusion

This pilot study is a proof of the concept that the use of RH or Reiki is as effective as manual manipulation PT in improving ROM in patients with painful shoulder limitation when evaluated immediately after a 10-minute treatment. The results suggest that it would be beneficial for physical therapists to be trained in RH or Reiki as well as PT so that they could reduce the need for manual work on patients, at least in cases of shoulder limitations. However, further research is required in which patients are reevaluated over longer time periods to determine whether the healing effect of a 10-minute RH or Reiki session is sustained at least as long as for a 10-minute PT session. The degree of increased effectiveness of longer or repeated treatments of RH, Reiki, or PT would also need to be compared.

## Figures and Tables

**Table 1:** Medical diagnoses of experimental participants.

Number of patients	SLAP tear	Torn RC	Arthritis	Impingement	Bone spur	Injury	Capsulitis	Bursitis	Unknown	X-ray/MRI
78	1	22	18	4	2*	14	2**	2	16	55

*One case was in combination with torn rotator cuff.

**Both cases were in combination with torn rotator cuff.

SLAP: superior labrum, anterior to posterior.

**Table 2:** Group distributions of medical diagnoses and surgeries.

Diagnosis/surgery	Number of Control groups	Number of Sham groups	Number of PT groups	Number of Reiki groups	Number of RH groups
SLAP	0	0	0	0	1
Torn RC	4	4	5	5	4
Arthritis	3	5	3	3	4
Impingement	1	1	0	1	1
Bone spur	0	0	1	1	0
Injury	3	3	3	3	2
Capsulitis	1	0	0	1	0
Bursitis	1	1	0	0	0
Unknown	3	1	4	2	6
Shoulder surgery	3	3	2	5	3

**Table 3:** Mean pre- and post-ROM averaged over palms up and down and left and right arms.

Group	Mean ROM Pre ± SD (degrees)	Mean ROM Post ± SD (degrees)	Difference (Post − pre)	*N*
Control	44.96 ± 28.46	47.97 ± 30.78	3.01	15
Sham	48.13 ± 31.63	48.75 ± 31.56	0.62	15
PT	51.47 ± 35.49	63.63 ± 46.99	12.16	16
Reiki	24.85 ± 8.35	44.58 ± 27.39	19.73	15
RH	47.81 ± 32.31	74.17 ± 58.02	26.36	17

**Table 4:** Mean pre- and postpain scores.

Group	Mean pain Pre ± SD	Mean pain Post ± SD	Difference (Post − pre)	*N*
Control	46.4 ± 5.7	46.6 ± 5.4	0.2	15
Sham	53.2 ± 5.8	37.8 ± 5.4	−15.4	15
PT	52.1 ± 5.6	40.6 ± 5.2	−11.5	16
Reiki	41.3 ± 5.7	31.2 ± 5.4	−10.1	15
RH	46.1 ± 5.4	22.2 ± 5.1	−23.9	17
